# The Anorexia Nervosa: Genetics Initiative (ANGI): Australia and New Zealand join forces

**DOI:** 10.1186/2050-2974-3-S1-P10

**Published:** 2015-11-23

**Authors:** Martin Kennedy, Jennifer Jordan, Nick Martin, Tracey Wade, Grant Montgomery, Cynthia Bulik

**Affiliations:** 1 University of Otago, Christchurch, New Zealand; 2 Queensland Institute of Medical Research-Berghofer, Brisbane, Australia; 3 Flinders University, Bedford Park, Australia; 4 University of North Carolina at Chapel Hill, Chapel Hill, NC, USA; 5 The Karolinska Institute, Solna, Sweden

## Introduction

Anorexia nervosa (AN) is familial and heritable and the next logical step is to identify genes that influence risk. In the genome-wide association study (GWAS) era, for the study of complex traits such as eating disorders, large sample sizes are essential and necessitate global cooperation. The Anorexia Nervosa Genetics Initiative (ANGI) is a collaboration of researchers and clinicians from the United States, Sweden, Demark, and Australia and New Zealand who are collecting clinical information and DNA from 13,000 individuals with AN and controls. ANGI will unite with other global efforts to a goal of 25,000 participants with AN.

## Methods

We aim to recruit 2200 Australian and 300 New Zealand males and females aged 12+ years (Australia) and 14+ (New Zealand) with lifetime AN. Participants complete an online survey which collects phenotypic information and provide a blood sample. GWAS will be conducted in the USA.

## Results

ANGI is actively recruiting across New Zealand and Australia. Progress to date will be presented.

## Discussion

Large samples and international collaborations maximise the chances of answering fundamental questions about the biology of AN. Identifying genes contributing to risk and clarifying pathophysiological pathways may lead to better prevention and treatment of anorexia nervosa.

